# Editorial: Welfare and Stressors in Fish: Challenges Facing Aquaculture

**DOI:** 10.3389/fphys.2020.00162

**Published:** 2020-02-25

**Authors:** Juan Antonio Martos-Sitcha, Juan Miguel Mancera, Patrick Prunet, Leonardo Julián Magnoni

**Affiliations:** ^1^Department of Biology, Faculty of Marine and Environmental Sciences, Instituto Universitario de Investigación Marina (INMAR), Campus de Excelencia Internacional del Mar (CEIMAR), University of Cádiz, Cádiz, Spain; ^2^INRAE, UR1037, Laboratoire de Physiologie et de Génomique des Poissons (LPGP), Rennes, France; ^3^Interdisciplinary Centre of Marine and Environmental Research (CIIMAR), University of Porto, Matosinhos, Portugal

**Keywords:** fish welfare, environmental stressors, dietary imbalance, handling, fish monitoring devices, stress-reducing agents, HPI axis, energy balance

Aquaculture production is in a process of expansion as demand increases globally. Nevertheless, the intensive production of fish requires carefully monitored and controlled environments that, if not adhered to, may lead to stressed animals, compromising their health and survival (Ashley, 2007). Efforts to improve welfare and to reduce mortality in fish aquaculture are reflected in European Directive 2010/63/EU, and its implementation has been evaluated by Toni et al. (2018). Improving the welfare of farmed fish (e.g., by reducing stress) can result in enhanced productivity. Therefore, applying correct management protocols is important for the economic success of this industry. In addition, there is now an increased awareness among the public and the scientific community of the importance of understanding the physiological and behavioral bases of stress responsiveness and welfare in fish (Conte, 2004; Huntingford et al., 2006; Braithwaite and Ebbesson, 2014; Castanheira et al., 2017). Ethical aspects of the use of fish for aquaculture, research as well as in fisheries have also been considered (Huntingford and Kadri, 2009; Bovenkerk and Meijboom, 2013).

Intensive production may involve decreased environmental quality, including increased fish density and the appearance of production-related diseases, which are challenges faced by aquaculture. Domestication could lead to lower stress levels of the fish population for certain aquaculture environment. However, domestication may impair stress coping when fish experience a change in that environment. Therefore, the effects of multiple concurrent stressors, the process of domestication and the mechanisms associated with stress responsiveness are aspects that still need to be investigated in cultured fish. For these reasons, this Research Topic was aimed at expanding general knowledge on the physiological responses of cultured aquatic animals to current practices as well as finding alternatives to improve conditions.

Over the past few decades we have seen an increased number of studies characterizing the stress response in fish, however, the information for other aquatic organisms, such as the cephalopods, is limited. Several Octopodidae species have great potential for aquaculture. Unfortunately, the lack of stress-related biomarkers in this taxon presents an obstacle to evaluate maintenance conditions. Barragán-Méndez et al. assessed physiological responses related to fishing capture of *Eledone moschata, E. cirrhosa*, and *Octopus vulgaris* as a first step toward studying the physiology of stress. The authors reported information on energy mobilization in response to capture, which returned to pre-stress values within the first 24 h.

Cortisol is believed to be the main hormone mediating the physiological stress response in vertebrates. Sustained swimming activity at optimal speeds is associated with improved growth and lower secreted cortisol levels in various fish species. Palstra et al. demonstrated that best growth in zebrafish (*Danio rerio*) occurs at the optimal swimming speed and is associated with increased cortisol levels. However, the response was not directly mediated by the glucocorticoid receptor (Gr), suggesting that cortisol is not the main determinant of exercise-enhanced growth in this species. This study showed that 36 genes in white skeletal muscle, involved in transcriptional regulation and protein ubiquitination, play a major role in the growth-promoting effects of exercise in fish.

In addition to swimming, other factors impact fish homeostasis and may act as stressors. Among them water dissolved oxygen levels and stocking density are relevant factors under intensive aquaculture production. Therefore, physiological responses and the molecular mechanisms unchained by stressors need to be clarified to establish optimal animal welfare and maximize efficiency, especially when multiple factors may coexist. Magnoni et al. investigated the response of rainbow trout (*Oncorhynchus mykiss*) subjected to both long-term environmental hypoxia and dietary electrolyte-imbalance followed by an acute stressor. In spite of a decrease in feed intake, stress markers were not altered by hypoxia. The dietary challenge profoundly affected fish homeostasis, evoking oxidative stress and impaired immune status, which led to stress and potentiating the negative effect of the acute stressor, suggesting a synergistic effect. Furthermore, Hernández-Perez et al. showed that high stocking density in rainbow trout alters liver metabolism, a response also modulated by circadian rhythms. This study confirmed that high stocking density induces a stress response that is finely, differently and time-dependently regulated by glucocorticoid pathways. The circadian oscillator located in the liver was affected by the stressor, modulating the energy partitioning. Likewise, Martos-Sitcha, Simó-Mirabet et al. demonstrated by using metabolic and transcriptomic approaches that moderate hypoxia in gilthead seabream (*Sparus aurata*) produces a hypometabolic state with a negative impact on feed intake and growth rate, effects that were intensified with high stocking density. The response was accompanied by an improvement in the feed efficiency, an enhancement in the O_2_-carrying capacity, as well as a differential regulation of metabolic and stress markers. This was shown by several physiological hallmarks responding to the stressor across several organs, suggesting a different contribution of each tissue to the allostatic load.

The fish skin is a multifunctional organ with highly relevant physiological roles. Kulczykowska summarizes the current knowledge of the skin function as a cutaneous stress response system, where cortisol, melatonin, and derived peptides act together to protect the organism against unfavorable conditions. The study of the various skin functions along with the impact of environmental and biotic factors is an exciting research area rapidly expanding due to its relevance in cultured fish. Sanahuja et al. investigated the response of the skin mucus proteome in the gilthead seabream exposed to changes in water temperature. Authors found that proteins associated with a stress response were up-regulated in fish exposed to low temperatures. However, proteins related to metabolic activity were down-regulated in response to cold, evidencing depressed skin metabolism. Results show a partial loss of mucus functionality under chronic cold exposure, which may affect fish welfare under farming conditions. Interestingly, parameters measured in the skin mucus can be used in a non-invasive approach to assess fish welfare.

Detrimental effects caused by adverse rearing conditions could be alleviated by therapeutic strategies, which is a promising area of research aimed at improving aquaculture production. The use of different amino acids in fish diets may be an interesting tool to mitigate stress responses, although the effect may be dependent on the species, the stressor and quantity supplemented. Azeredo et al. investigated the effects of tryptophan (Trp) dietary supplementation in Senegalese sole (*Solea senegalensis*) held at high stocking densities. The study showed that fish fed a Trp supplemented diet were better prepared to cope with a biotic challenge, showing a decreased cumulative mortality. The effects of dietary Trp supplementation have been investigated in the meagre (*Argyrosomus regius*) as well, a cultured species with increasing importance in Southern Europe. In this study Asencio-Alcudia et al. showed that the expression of several genes related to the immune response were up-regulated in fish fed the amino acid supplemented diet, suggesting its potential to improve tolerance and/or alleviating acute response to handling stressors of this species.

Anesthetics could be used to reduce the negative effects of stressors associated with aquaculture, including transport. Jerez-Cepa et al. and Teles et al. investigated the effects of a sedative dose of clove oil (CO) and MS-222 on hallmarks of the HPI axis regulation, energy management, and oxidative stress in gilthead seabream after simulated transport and further recovery. Jerez-Cepa et al. showed that HPI axis response was modified at plasma level, with differences depending on the anesthetic employed. Gene-expression related to cortisol production in the head kidney matched with the increased plasma cortisol levels immediately after transport in CO-sedated fish, but these levels remained constant in MS-222-sedated fish. Differential changes in the energy management of carbohydrates, lipids, and amino acids were dependent upon the anesthetic employed. In addition, Teles et al. reported that the use of both CO and MS-222 interferes with fish antioxidant status. The expression levels of genes related to antioxidant response and cell-tissue repair were altered in several tissues, confirming that both sedatives may have long-term effects on fish defenses, although minimizing the stress associated with transport. These results are highly relevant to aquaculture considering that oxidative stress may increase the fishes' susceptibility to other stressors and to pathogens.

Essential oils (EOs) could be a promising tool to reduce the stress associated with aquaculture procedures, improving welfare. EOs may be used as sedatives/anesthetics by reducing oxidative stress and/or boosting the immune response. Souza et al. reviewed the potential application of several EOs extracted from plants in fish subjected to several stressors. EOs have been reported to have a modulatory effect on the metabolic response of fish when included in the diet or added into the water. EOs displayed reduced adverse effects as compared to synthetic compounds, although adequate concentrations and chemotypes to be used need to be further investigated.

Vaccination is a therapeutic strategy widely used in aquaculture to improve fish health although this may involve handling stress, implying a compromise in the fishes' welfare. Whether this vaccination elicits stress responses and enhances the crosstalk between the immune and endocrine systems in the brain or pituitary is unclear. Liu et al. investigated the stress and immune responses in gilthead seabream exposed to two different vaccine routes. The authors report that no stress response was induced for both routes of vaccination in the brain or pituitary using gene expression analysis, although plasma cortisol response was linked to both procedures. However, the authors showed an alteration of corticotropin-releasing hormone-binding protein (*crhbp*) and glucocorticoid receptor *(gr)*, which suggests that the proteins coded by both genes could play a relevant role in the feedback regulation of HPI axis after vaccination.

Finally, technical solutions aiming to monitor fish welfare using less-invasive and non-lethal procedures by employing sentinel organisms are novel tools to be implemented in aquaculture. Martos-Sitcha, Sosa et al. described the design and validation of a reprogrammable and miniaturized device that can be attached to the operculum of gilthead seabream and European seabass (*Dicentrarchus labrax*). The authors reported how this device recorded and quantified the activity and respiratory frequency in fish kept in rearing tanks after its corresponding validation with swimming respirometry. Tagging the fish with this device was shown to have a minimal and transient impact, demonstrating that this miniaturized device could be a suitable tool when characterizing the physiological and behavioral responses of fish leading to improved performance and welfare in farmed species.

This Research Topic included several aspects related to the assessment of welfare in cultured fish, describing the stress response and the implementation of new procedures in order to decrease the negative effects of stressors existing in aquaculture practices. In addition, new devices to assess the wellbeing of relevant species are described. However, further studies will be required to better understand the mechanisms mediating stress responses and the possible strategies to reduce the impact of stressors associated with aquaculture.

## Author Contributions

JM-S, JM, PP, and LM contributed writing this editorial.

### Conflict of Interest

The authors declare that the research was conducted in the absence of any commercial or financial relationships that could be construed as a potential conflict of interest.
